# Post-activation Performance Enhancement in the Bench Press Throw: A Systematic Review and Meta-Analysis

**DOI:** 10.3389/fphys.2020.598628

**Published:** 2021-01-15

**Authors:** Michal Krzysztofik, Michal Wilk, Petr Stastny, Artur Golas

**Affiliations:** ^1^Institute of Sport Sciences, The Jerzy Kukuczka Academy of Physical Education in Katowice, Katowice, Poland; ^2^Department of Sport Games, Faculty of Physical Education and Sport, Charles University, Prague, Czechia

**Keywords:** ballistic exercise, post-activation potentiation (PAP), resistance training, explosive strength, strength training, power output (PO), sport performance

## Abstract

**Background:** Mechanical power output is recognized as a critical characteristic of an athlete with regard to superior performance during a competition. It seems fully justified that ballistic exercises, in which the external load is projected into a flight phase, as in the bench press throw (BPT), are the most commonly prescribed exercises for the development of power output. In addition, the muscular phenomenon known as post-activation performance enhancement (PAPE), which is an acute improvement in strength and power performance as a result of recent voluntary contractile history, has become the focus of many strength and conditioning training programs. Although the PAPE phenomenon is widely used in the upper-body training regimens, there are still several issues regarding training variables that facilitate the greatest increase in power output and need to be resolved.

**Objective:** The purposes of this meta-analysis were to determine the effect of performing a conditioning activity (CA) on subsequent BPT performances and the influence of different types of CA, intra-complex rest intervals, and intensities during the CA on the upper-body PAPE effect in resistance-trained men.

**Methods:** A search of electronic databases (MEDLINE, PubMed, and SPORTDiscus) was conducted to identify all studies that investigated the PAPE in the BPT up to August 2020. Eleven articles, which met the inclusion criteria, were consequently included for quality assessment and data extraction. All studies included 174 resistance-trained men [age: 25.2 ± 2.1 years; weight: 88.4 ± 7.5 kg; height: 1.82 ± 0.03 m; bench press (BP) relative strength: 1.31 ± 0.14 kg ± kg^−1^] as participants. Meta-analyses of standardized mean effect size (ES) between pre-CA mean and post-CA mean from individual studies were conducted using the random-effects model.

**Results:** The effect of PAPE in the BPT was small (ES = 0.33; *p* < 0.01). The BP exercise as a CA at an intensity of 60–84% one-repetition maximum (1RM) (ES = 0.43) induced slightly greater PAPE effect than a ballistic–plyometric (ES = 0.29) and a BP exercise at ≥85% 1RM and at >100% 1RM as well as a concentric-only BP (ES = 0.23 and 0.22; ES = 0.11, respectively). A single set (ES = 0.37) of the CA resulted in a slightly greater effect than a multiple set (ES = 0.29). Moderate rest intervals induced a slightly greater PAPE effect for intensity below 85% 1RM (5–7 min, ES = 0.48) than shorter (0.15–4 min, ES = 0.4) and longer (≥8 min, ES = 0.36) intra-complex rest intervals. Considering an intensity above 85% 1RM during the CA, a moderate rest interval resulted in a similar PAPE effect (5–7 min, ES = 0.3) compared with longer (8 min, ES = 0.29) intra-complex rest interval, whereas shorter rest intervals resulted in a negative effect on BPT performance (0.15–4 min, ES = −0.13).

**Conclusion:** The presented meta-analysis shows that performing a CA induces a small PAPE effect for the BPT performance in resistance-trained men. Individuals seeking to improve their BPT performance should consider preceding them with a single set of the BP exercise at moderate intensity (60–84% 1RM), performed 5–7 min before the explosive activity.

## Introduction

Mechanical power output is recognized as a critical characteristic of an athlete competing in explosive sport disciplines, such as sprints, jumps, and throws in athletics (Cormie et al., 2011; Haff and Nimphius, 2012). Ballistic exercises, where the external load is projected into a flight phase (e.g., throws), are the most commonly prescribed for the development of power output (Suchomel et al., 2018). They have been shown to produce greater velocity and power output than non-ballistic resistance exercises (Frost et al., 2010; Requena et al., 2011). The rationale for these increments is the requirement to accelerate throughout the entire concentric movement in order to project the load into the flight phase. Accordingly, the path of acceleration of the barbell is increased, requiring greater average vertical force to be applied to the load and achieving higher vertical velocities, resulting in greater power outputs (Newton et al., 1997; Lake et al., 2012), unlike non-ballistic resistance exercises that require the barbell to have zero momentum at the end of the concentric phase. Regarding the upper body, the use of the bench press throw (BPT) is recommended and is indicated as one of the most effective exercises for achieving improvements in power output of the upper body (Newton et al., 1997; Cormie et al., 2011; Sarabia et al., 2017; Sakamoto et al., 2018). Furthermore, the BPT has been associated with overall performance in different sport-specific tasks (Cronin and Owen, 2004; Sakamoto et al., 2018). Therefore, it seems reasonable to use the BPT as a means of developing and testing upper-body ballistic performance as the most effective exercise for power of the upper limbs.

A muscular phenomenon known as post-activation potentiation, which is an acute improvement in strength and power performances as a result of the recent voluntary contractile history (Tillin and Bishop, 2009; Seitz and Haff, 2016), has become the focus of many strength and conditioning training programs. In training, increased force and power output production is induced by a potentiation complex consisting of a conditioning activity (CA) [i.e., bench press (BP)], followed by an explosive activity with a similar movement structure (i.e., BPT) (Gołaś et al., 2016; Seitz and Haff, 2016). The primary mechanism responsible for this effect is the phosphorylation of the myosin regulatory light chain (MRLC) that leads to an increase in calcium sensitivity of the actomyosin complex (Tillin and Bishop, 2009; Blazevich and Babault, 2019). Since most studies evaluating the post-activation potentiation effect did not provide confirmatory evidence, such as muscle twitch force assessment considered to reflect the magnitude of the post-activation potentiation, recently, an alternative term, referred to as post-activation performance enhancement (PAPE), has been proposed (Cuenca-Fernández et al., 2017; Blazevich and Babault, 2019; Boullosa et al., 2020). The rationale for this phenomenon may be related to the residual of the post-activation potentiation in its earliest stages after a CA and other mechanisms, such as an increase in muscle temperature, fiber water content, and muscle activation (Blazevich and Babault, 2019). Nevertheless, the occurrence of performance improvement is particularly dependent on the optimal relationship between potentiation and fatigue (Rassier and Macintosh, 2000). The most significant condition of achieving performance enhancement is that potentiation induced by the above-mentioned mechanisms has to exceed the fatigue produced at the same time.

The efficiency of various CA has been examined by numerous authors, which suggest that a wide range of sport-specific tasks, such as jumping, throwing, and running, can be enhanced through the PAPE phenomenon (Wilson et al., 2013; Seitz and Haff, 2016). Accordingly, numerous studies have investigated the different variations of CA, mainly by using different types of exercises, with varied intensity (external loads), volume (number of sets and repetitions), as well as the duration of the intra-complex rest intervals (the period between the CA and the subsequent exercise). Among other factors that can affect power output, training experience and individual strength level should be considered (Docherty and Hodgson, 2007; Wilson et al., 2013; Seitz and Haff, 2016). For example, Chiu et al. (2003) compared the potentiation response in competitive athletes and recreationally trained participants and reported significantly greater explosive performance enhancement in competitive athletes. This is supported by findings from Wilson et al. (2013) who indicated that athletes with more than 3 years of resistance training experience are able to express greater potentiation levels than those with 1 year of resistance training experience or untrained participants. In regard to the strength level, Seitz and Haff (2016) reported that stronger participants (≥1.35 kg/bw in the BP and ≥1.75 in the back squat) exhibited greater PAPE effect than their weaker counterparts (<1.35 kg/bw in the BP and <1.75 kg/bw in the back squat).

Furthermore, previous meta-analysis revealed that far fewer studies have examined the magnitude of the PAPE effect in the case of upper body than lower body (Wilson et al., 2013; Seitz and Haff, 2016). Moreover, to the best of our knowledge, no meta-analysis has been conducted to separately investigate the upper-body PAPE effect, whereas there are such for the lower body (Gouvêa et al., 2013; Dobbs et al., 2019). The creation of a separate meta-analysis dedicated to the upper-body PAPE effects seems to be especially valuable, as there is evidence that different results have been obtained for the lower limbs using the same variables of the CA, so it could be speculated that inducing the PAPE effect requires different approaches for the upper and lower bodies (Kilduff et al., 2007; Farup and Sørensen, 2010). Despite that, there are significant data to quantify the upper-body PAPE effect induced by a variety of CA. For example, Brandenburg (2005) investigated how different intensities [50 vs. 75 vs. 100% five-repetition maximum (5RM)] of the BP exercise CA influence subsequent BPT performance. Neither CA showed an improvement in performance. In contrast, Liossis et al. (2013) found that a single set of the BP exercise performed at 65 and 85% one-repetition maximum (1RM) significantly augments post-activation BPT performance; however, it depends on the time of the intra-complex rest interval. In regard to higher intensity, an 8 min rest interval is necessary to achieve potentiation, whereas for lower intensities, a 4 min rest interval seems sufficient. Moreover, Bevan et al. (2009) reported that three sets of three BP repetitions at 87% 1RM augment BPT performance when performed 8 min prior to the explosive activity. Briefly, despite the studies that examined the influence of several training variables used during the CA on the magnitude of upper-body PAPE effect, there is no consensus in regard to the optimal level of these variables.

Therefore, there is a need for additional research to evaluate the efficacy of various upper-body CA that may help in the selection of the most appropriate training variables to elicit the PAPE effect. Accordingly, the purposes of this meta-analysis were to determine (1) the effects of performing a CA on subsequent BPT performances and (2) the influence of different types of CA, intra-complex rest intervals, and intensities during the CA on the PAPE effect. The hypotheses of this investigation were: (1) improvements in the BPT performances will be observed after a CA and (2) the type of a CA, the intensity used during the CA, and the intra-complex rest intervals will elicit different PAPE responses.

## Methods

The methodology of this systematic review was planned according to the Preferred Reporting Items for Systematic Reviews and Meta-Analyses (PRISMA) guidelines (Moher et al., 2009).

### Literature Search

A search of electronic databases was conducted to identify all studies that investigate the post-activation potentiation and PAPE phenomenon up to 1st of August 2020. As a prerequisite, all studies were performed in healthy populations of athletes including male adults (>18 years). Search terms were combined by Boolean logic (AND, OR) in MEDLINE, PubMed, and SPORTDiscus. The search was undertaken using the following keyword combinations in the English language: “bench press throw,” “PAP,” “post activation potentiation,” “PAPE,” “post activation performance enhancement,” “conditioning contraction,” and “conditioning activity.” Additionally, the reference lists and citations of the selected articles were scanned using Google Scholar to find additional articles. The authors of published papers were also contacted directly if crucial data were not reported in original papers.

### Inclusion and Exclusion Criteria

Research studies investigating the effects of different CAs on subsequent BPT performance enhancement were the primary focus of the literature search. A total of 77 studies were initially identified for further scrutiny.

**Figure 1:**
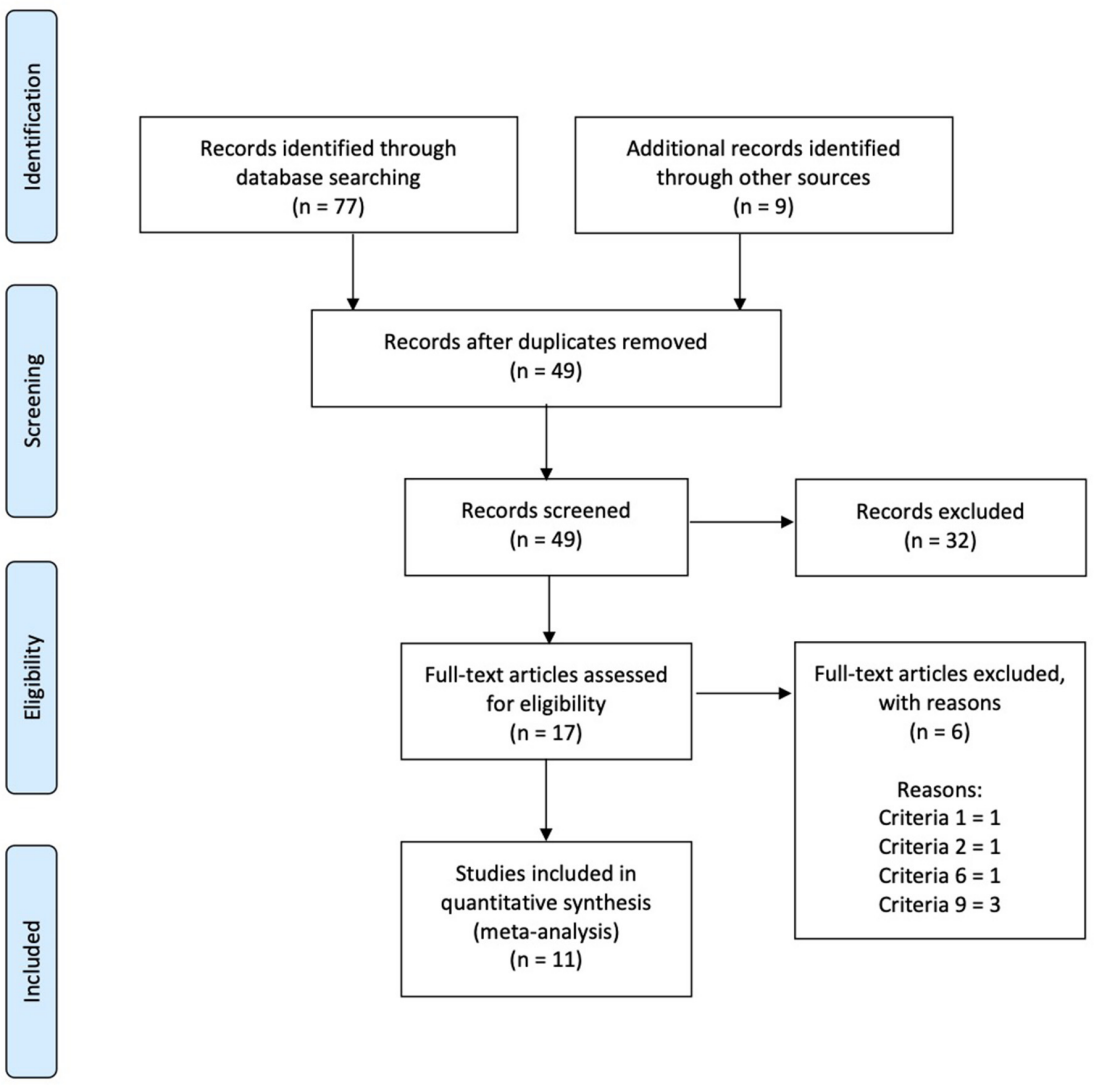
PRISMA flow diagram detailing the study inclusion process.

The following inclusion criteria were used to select articles for the meta-analysis: (1) high reliability and validity tests evaluating BPT performance, (2) the participants were able to perform the BP exercise with a load exceeding their body mass, (3) a pre-CA test was carried out at baseline before the CA, (4) a warm-up was performed prior to completing the pre-CA test, (5) the pre- and post-CA tests and the CA were performed during the same experimental session, (6) the BPT was performed on a Smith machine, (7) the study did not use any electrically elicited stimuli during the CA, (8) the study did not use any isokinetic equipment during both the performance tests and the CA, and (9) the study reached at least 4 points in the methodological quality criteria Table 1. After critically analyzing the initial studies collected with the above criteria, a cohort of 11 studies was selected for further analysis (Figure 1, Tables 2, 3).

**Table 1:** List of criteria for assessment of the methodological quality of studies.

**No**.	**Item**	**Score**
(1)	Sample description:	0 or 1
	Properties of the subjects (age, weight, height, and sex)	
	Definition of the population (athlete, recreationally trained, active subjects, and untrained)	
	Training status and resistance training experience	
(2)	Procedure description:	0 or 1
	Detailed description of the protocol (randomized order of evaluations, standard testing conditions, and assessment of different CAs on separate days)	
	Detailed description of the CA (type, volume and intensity, rest intervals)	
	Developed a familiarization period with the exercises used during the experimental protocol (last few weeks or last days)	
(3)	Intervention:	0 or 1
	Defined exercise technique (bar position, supervision of a strength coach)	
	Defined loading conditions (Smith machine)	
(4)	Measurement system, data collection, and data analysis:	0 or 1
	Device description (brand of the product, model, manufacturer)	
	Defined sampling frequency of the product	
	Information about the reliability and validity of the measuring system	
	Specification of software for recording and analyzing data	
(5)	Results detailed:	0 or 1
	Measure of the central tendency	
	Amount of variation or dispersion from the average	

**Table 2:** Assessment of the risk of bias in nonrandomized studies of interventions.

**Study**	**Bias due to confounding**	**Bias in selection of study participants**	**Bias in measurement classification of interventions**	**Bias due to deviations from intended interventions**	**Bias due to missing data**	**Bias in measurement of outcomes**	**Bias in selection of reported results**	**Overall risk of bias**
Brandenburg (2005)	Low	Moderate	Moderate	Moderate	Low	Moderate	Moderate	Moderate
Bevan et al. (2009)	Low	Low	Moderate	Low	Moderate	Moderate	Moderate	Moderate
Esformes et al. (2011)	Low	Moderate	Moderate	Moderate	Serious	Moderate	Moderate	Serious
Farup and Sørensen (2010)	Moderate	Moderate	Moderate	Low	Serious	Moderate	Serious	Serious
Kilduff et al. (2007)	Low	Low	Moderate	Low	Moderate	Moderate	Moderate	Moderate
Krzysztofik et al. (2020b)	Moderate	Low	Moderate	Moderate	Moderate	Moderate	Moderate	Moderate
Liossis et al. (2013)	Low	Moderate	Moderate	Low	Moderate	Moderate	Moderate	Moderate
Tsoukos et al. (2019)	Low	Low	Low	Low	Low	Low	Low	Low
Tsoukos et al. (2020)	Low	Low	Low	Low	Low	Low	Low	Low
Ulrich and Parstorfer (2017)	Low	Low	Moderate	Low	Moderate	Moderate	Moderate	Moderate
West et al. (2013)	Low	Low	Moderate	Moderate	Moderate	Moderate	Moderate	Moderate

*ROBINS-I, Risk of Bias in Nonrandomized Studies of Interventions. The categories for risk of bias for each domain are “low risk,” “moderate risk,” “serious risk,” and “critical risk” of bias and “no information”*.

*The overall risk of bias was classified as low if all domains were at low risk of bias, as moderate if all domains were at low/moderate risk of bias, and as serious if at least one domain was at serious risk of bias but not at critical risk of bias in any domain. The risk of bias was classified as critical if at least one domain was at critical risk of bias. No information was defined if there is a lack of information in one or more key domains of bias*.

**Table 3:** Studies meeting the inclusion criteria.

**Article**	**Participants**			**Conditioning activity**		**Intra-complex rest intervals**	**Potentiated exercise**	**Quality**
	***n***	**Training experience**	**Strength level**	**Exercise**	**Volume and load**			
Brandenburg (2005)	9	>1 year	1.46 kg/bw	Bench press	(a) 1 set of 5 repetitions at 100% 5RM	4 min	Concentric-only bench press throw at 45% 1RM	5
					(b) 1 set of 5 repetitions at 75% 5RM			
					(c) 1 set of 5 repetitions at 50% 5RM			
Bevan et al. (2009)	26	>2 years	1.35 kg/bw	Bench press	3 sets of 3 repetitions at 87% 1RM with 4 min rest	15 s, 4, 8, 12, 16, 20, 24 min	Bench press throw at 40% 1RM	4
Esformes et al. (2011)	10	>2 years	1.14 kg/bw	Bench press	(a) 7 s isometric	12 min	Bench press throw at 40% 1RM	5
					(b) 1 set of 3 concentric repetitions at 3RM			
					(c) 1 set of 3 eccentric repetitions at 3RM			
					(d) 1 set of 3 eccentric–concentric repetitions at 3RM			
Farup and Sørensen (2010)	8	>1 year	1.4 kg/bw	Bench press	5 sets of 1RM with 5 min inter-set rest period	1–21 min	Bench press throw at 30% 1RM	4
Kilduff et al. (2007)	23	>3 years	1.27 kg/bw	Bench press	1 set of 3RM	15 s, 4, 8, 12, 16, 20 min	Bench press throw at 40% 1RM	4
Krzysztofik et al. (2020b)	32	>3 years	1.54 kg/bw	Bench press	(a) 2 sets of 2 eccentric repetitions at 90% 1RM	5 min	Bench press throw at 30% 1RM	4
					(b) 2 sets of 2 concentric repetitions at 90% 1RM			
					(c) 2 sets of 2 eccentric repetitions at 110% 1RM			
					(d) 2 sets of 2 eccentric repetitions at 130% 1RM			
Liossis et al. (2013)	9	>6 months	1.09 kg/bw	Bench press	(a) 5 repetitions at 65% 1RM	4 and 8 min	Bench press throw at 30% 1RM	5
					(b) 5 repetitions at 85% 1RM			
Tsoukos et al. (2019)	10	>3 years	1.26 kg/bw	Bench press	(a) 40% 1RM until mean velocity dropped to 90% of the peak attained	45 s, 2, 4, 6, 8, 10, 12 min	Bench press throw at 30% 1RM	5
					(b) 40% 1RM until mean velocity dropped to 70% of the peak attained			
					(c) 60% 1RM until mean velocity dropped to 90% of the peak attained			
					(d) 60% 1RM until mean velocity dropped to 70% of the peak attained			
					(e) Control in which participants did not perform any CA			
Tsoukos et al. (2020)	11	>3 years	1.29 kg/bw	Bench press	(a) 80% 1RM until mean velocity dropped to 90% of the peak attained	45 s, 2, 4, 6, 8, 10, 12 min	Bench press throw at 30% 1RM	5
					(b) 80% 1RM until mean velocity dropped to 90% of the peak attained			
					(c) Control in which participants did not perform any CA			
Ulrich and Parstorfer (2017)	16	>1 year	1.18 kg/bw	Bench press and plyometric push-ups	(a) 3 repetitions at 80% 1RM	1, 4, 8, 12, 16 min	Bench press throw at 30% 1RM	5
					(b) 3 eccentric repetitions at 120% 1RM			
					(c) 10 repetitions of plyometric push-ups			
West et al. (2013)	20	>2 years	1.48 kg/bw	Bench press and bench press throw	(a) 3 sets of 3 repetitions at 87% 1RM	8 min	Bench press throw at 30% 1RM	5
					(b) 3 sets of 3 repetitions of bench press throw at 30% 1RM			

*RM, repetition maximum; CA, conditioning activity*.

### Methodological Quality of Included Studies

Study quality was evaluated by a modified standard procedure from Soriano et al. (2015) (Table 1). Each study was read and ranked by three independent investigators (MK, MW, and AG), with a larger number indicating better quality. For each question, 1 point was awarded if the study met the standard. If an insufficient description or data were provided to analyze a specific question, 0 points were awarded. The score was then tallied for each of the questions, with the highest score possible equaling 5 out of 5 points. Moreover, the Risk of Bias in Nonrandomized Studies of Interventions (Sterne et al., 2016) was used by two independent investigators (MK, PS) to jointly create an overall risk of bias score (“low risk,” “moderate risk,” “serious risk,” and “critical risk” of bias and “no information”) (Table 2). Nearly three–fourths of the studies were considered to be at moderate or serious risk of bias mainly due to the lack of a control condition trial, recruitment of a small group of participants or not reported sample size power analysis, and measure of the potentiation effect after a single intra-complex rest interval.

Then, due to the fact that the PAPE effect can be influenced by numerous factors (Seitz and Haff, 2016), independent variables were grouped into the following categories: (1) potentiation complex characteristics including the type of the CA and its intensity (BP at supramaximal intensity: >100% 1RM, high intensity: 85–100% 1RM, moderate intensity: 60–85% 1RM, ballistic–plyometric exercise, concentric-only exercise), (2) volume of the CA (single set and multiple sets), and (3) intra-complex rest intervals depending on the intensity of exercise (0.3–4, 5–7, ≥8 min; for <85% and ≥85% 1RM). The mean agreement was calculated by an intra-class correlation coefficient (ICC), and for the present study, a 0.94 was reached. For such coding methods, a mean agreement of 0.90 is generally accepted as an appropriate level of reliability (Hedges and Olkin, 2014).

### Analysis and Interpretation of Results

Effect sizes (ESs) were used to obtain standardized measurements of the effect of the CA on the outcome variable. The ES is a standardized value that allows the determination of the magnitude of the differences between groups or experimental conditions (Thomas and French, 1986). The ESs were calculated using Hedges and Olkin's *g* (2014) as follows (Eq. 1):

(1) $$\begin{array}{r} \text{ES }=\;g\frac{\left(M_{post}-M_{pre}\right)}{SD_{pooled}} \end{array}$$

where *M*_*post*_ is the mean of the performance test completed after the CA, *M*_*pre*_ is the mean of the performance test completed before the CA, and *SD*_*pooled*_ is the pooled standard deviation of the measurements (Eq. 2):

(2) $$\begin{array}{r} SD_{pooled}=\frac{\left(\left(n_{1}-1\right)\times SD_{1}^{2}+\left(n_{2}-1\right)\times SD_{2}^{2}\right)}{\left(n_{1}+n_{2}-2\right)} \end{array}$$

where $SD_{1}^{2}$ is the standard deviation of the performance test completed before the CA, and $SD_{2}^{2}$ is the standard deviation of the performance test completed after the CA.

The ES should be corrected for the magnitude of the sample size of each study because the absolute value of the ES is over-estimated in small sample sizes (Hedges and Olkin, 2014). Therefore, a correction factor was calculated as follows (Eq. 3) (Hedges and Olkin, 2014):

(3) $$\begin{array}{r} Correction\;factor=1-\frac{3}{4\left(n_{1}+n_{2}-2\right)-1}. \end{array}$$

This method was chosen because it was recommended for calculation of the ES controlled in pre-test and post-test design studies in meta-analyses based on simulation results showing its superior properties with respect to bias, precision, and robustness to heterogeneity of variance compared with other methods (Morris, 2008).

The *corrected ES* was then calculated as follows (Eq. 4):

(4) $$\begin{array}{r} Corrected\;ES=g\times correction\;factor. \end{array}$$

The magnitude of the ES was considered as trivial (>0.2), small (0.2–0.50), moderate (0.50–0.80), and large (>0.80) according to the interpretation proposed by Cohen (2013) for sports training research. The *I*^2^ statistic was used to calculate heterogeneity among studies (Higgins and Thompson, 2002) and was classified as low (<25%), moderate (25–50%), or high (>75%). Heterogeneity was indicated if the *Q* statistic reached a significance of *p* < 0.05 and sampling error accounted to <75% of observed variance (Hedges and Olkin, 2014). Statistical analyses were carried out with the OpenMetaAnalyst version 10.12 (http://www.cebm.brown.edu/openMeta/).

## Results

**Figure 2:**
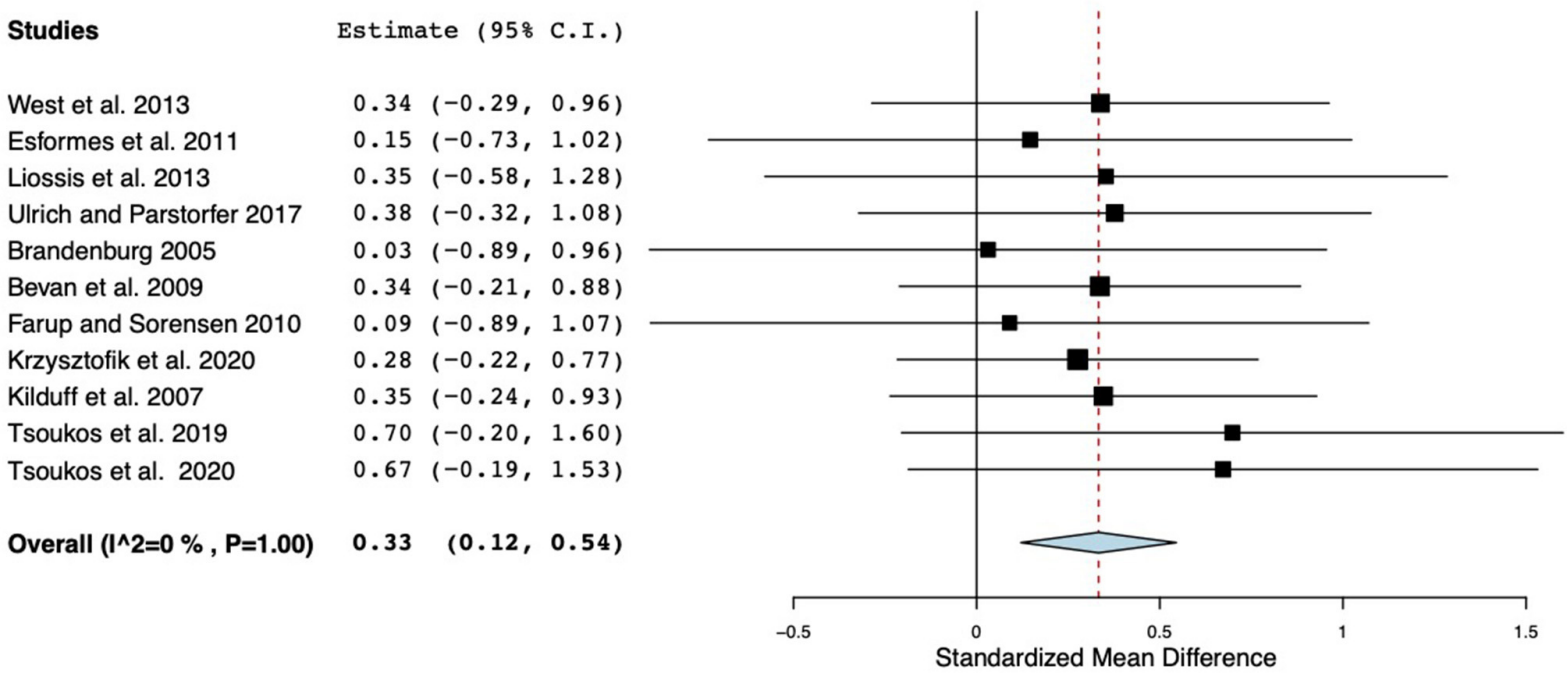
Forest plot of changes in the bench press throw performance.

Overall ES and moderating variables are presented in Table 4. The PAPE effect was small for the BPT performances (ES = 0.33; *p* < 0.01) and showed no heterogeneity (*Q*_10_ = 2.12; *p* = 1, *I*^2^ = 0%) (Figure 2). The analysis of various types of the CA revealed that a BP at an intensity of 60–84% 1RM (ES = 0.43) induced slightly greater PAPE effect than a ballistic–plyometric (ES = 0.29) and other resistance exercises at ≥85% 1RM and >100% 1RM (ES = 0.29, 0.23, and 0.22, respectively), whereas the concentric-only CA produced the lowest effect (ES = 0.11). In the case of the volume used during the CA, a single set (ES = 0.37) of the CA resulted in a slightly greater effect than a multiple set (ES = 0.29).

**Table 4:** Overall ES and moderating variables.

** Overall**		**Mean ± SD (95% CI)**	***N***
		**0.33 ± 0.2 (0.12–0.54)**	**174**
**Type of CA**			
	Ballistic–plyometric	0.29 ± 0.01 (−0.18–0.75)	36
	Concentric-only	0.11 ± 0.03 (−0.32–0.54)	42
	60–84% 1RM	0.43 ± 0.25 (0.05–0.81)	55
	85–100% 1RM	0.23 ± 0.14 (0.00–0.47)	137
	>100% 1RM	0.22 ± 0.09 (−0.18–0.62)	48
**Volume of CA**			
	Single set	0.37 ± 0.23 (0.08–0.67)	88
	Multiple set	0.29 ± 0.14 (−0.01–0.59)	86
**Intra-complex rest interval at** ** <85% 1RM**			
	0.15–4 min	0.4 ± 0.22 (0.02–0.77)	55
	5–7 min	0.48 ± 0.12 (0.02–0.94)	37
	≥8 min	0.36 ± 0.31 (−0.05–0.78)	46
**Intra-complex rest interval at** **≥85% 1RM**			
	0.15–4 min	−0.13 ± 0.15 (−0.47–0.21)	66
	5–7 min	0.3 ± 0.03 (−0.06–0.67)	58
	≥8 min	0.29 ± 0.15 (−0.05–0.6)	96

*Mean ± standard deviation (SD); N, number of participants; 1RM, one-repetition maximum; CA, conditioning activity*.

**Figure 3:**
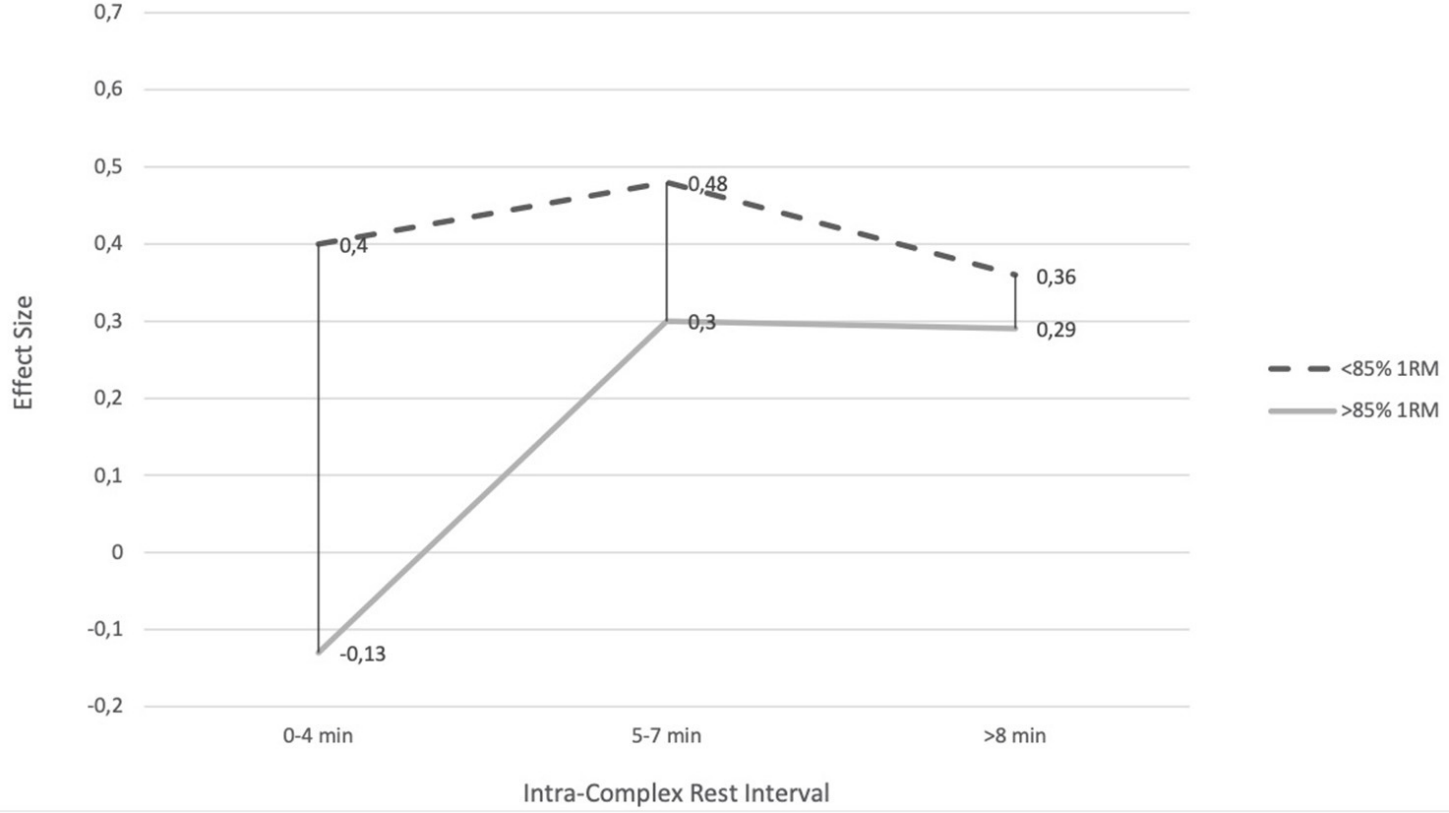
Bench press throw performance after different rest periods depending on the intensity of the conditioning activity.

With respect to the different intra-complex rest intervals in relation to the applied intensity of the CA, moderate rest intervals induced a slightly greater PAPE effect for intensity below 85% 1RM (5–7 min, ES = 0.48) than shorter (0.15–4 min, ES = 0.4) and longer (≥8 min, ES = 0.36) intra-complex rest intervals. Considering an intensity above 85% 1RM during the CA, a moderate rest interval resulted in a similar PAPE effect (5–7 min, ES = 0.3) compared with longer (≥8 min, ES = 0.29) intra-complex rest intervals, whereas shorter rest intervals resulted in a negative effect on BPT performance (0.15–4 min, ES = −0.13) (Figure 3).

## Discussion

The results of the meta-analysis show that performing a CA augments subsequent BPT performance in resistance-trained men. While the pooled effects were small, it should be taken into account that even a slight performance enhancement may be the worthwhile difference between winning and losing in sport disciplines or events requiring explosive strength of the upper limbs (Pyne et al., 2009; Grgic et al., 2019). The PAPE effect has been the subject of previous meta-analysis, yet there was no analysis performed separately for the upper body, although some studies indicate that the enhancement may differ between the upper and lower limbs (Kilduff et al., 2007; Farup and Sørensen, 2010). The results of the current study indicate that optimal BPT performance enhancement may be reached within 5–7 min after a single set of the BP exercise performed at moderate intensity (60–84% 1RM).

The present data suggest that the BP exercise performed at moderate intensity (60–84% 1RM) as the CA is slightly more effective (ES = 0.43) than ballistic–plyometric exercises (i.e., BPT at 30% 1RM or body weight plyometric push-ups; ES = 0.29) and the BP at high- and supramaximal intensities (ES = 0.23 and 0.22, respectively) in enhancing performance of subsequent BPTs. On the other hand, the concentric-only BP appears to be the least effective (ES = 0.11). These findings are consistent with those provided by Maloney et al. (2014) who indicated that high-intensity resistance exercises are more effective in inducing the PAPE effect than ballistic exercises. However, it has to be mentioned that only one study regarding the upper body was included in a review by Maloney et al. (2014); therefore, these results should be considered primarily for lower-body potentiation effects. Nevertheless, the authors highlighted that ballistic CA produces performance improvements in the range from 2 to 5%. The augmentation of performance after ballistic–plyometric CA may be related to the lower threshold of motor unit recruitment than during slower, ramped contractions (Ivanova et al., 1997; Van Cutsem et al., 1998), along with less fatigue than after traditional heavy loaded resistance exercises (Seitz and Haff, 2016). Furthermore, the advantage of ballistic–plyometric CA is the lack of cumbersome equipment requirements; thus, they can be employed as part of a warm-up routine before competition (Krzysztofik and Wilk, 2020).

The data from the current study indicated slight differences between single and multiple sets of the CA (ES = 0.37 vs. 0.29). While the meta-analysis by Wilson et al. (2013) as well as Seitz and Haff (2016) (ES = 0.24 vs. 0.66 and ES = 0.24 vs. 0.69, respectively) noticed significant differences between CA volume (single vs. multiple sets). Nonetheless, Seitz and Haff (2016) pointed out that in the case of individuals with higher strength levels, a greater effect appears after a single set (ES = 0.44) than a multiple set of the CA (ES = 0.21). Seitz and Haff (2016) grouped participants as strong, when the BP to body weight ratio exceeded ≥1.35 kg/bw, what is close to the average strength level of participants included in this meta-analysis (1.31 kg/bw). Despite that, multiple set CA may produce greater fatigue than a single set CA; it may not apply to stronger individuals who are more fatigue resistant (Chiu and Barnes, 2003). Moreover, it seems that multiple sets did not provide any additional benefits compared with a single set CA that seems to be sufficient to induce similar BPT performance enhancement in stronger individuals.

With respect to the intra-complex rest interval, regardless of the used intensity during the CA, the greatest effect has been observed after a 5–7 min rest interval (ES = 0.48). In regard to the intensity below 85% 1RM, the greatest effect has been observed after 5–7 min (ES = 0.48), followed by 0.15–4 min (ES = 0.4) and slightly smaller after ≥8 min rest interval (ES = 0.36). Considering an intensity above 85% 1RM during the CA, a similar effect was observed after 5–7 (ES = 0.3) and ≥8 min (ES = 0.29) intra-complex rest intervals, whereas 0.15–4 min rest interval resulted in a negative effect (ES = −0.13). These results are consistent with the observations of Seitz and Haff (2016) who showed that the greatest PAPE effect is achieved after the same length intra-complex rest intervals (5–7 min, ES = 0.49). A plausible explanation for these findings is the concomitant fatigue and potentiation induced by the CA. Thus, it can be concluded that a 5–7 min intra-complex rest interval seems optimal for improving explosive performance. The lack of improvements in 0.15–4 min after the CA at an intensity above 85% 1RM may be associated with greater fatigue that exceeds potentiation. In regard to the CA performed at an intensity below 85% 1RM, the opposite situation may occur. Furthermore, these findings may be associated with the muscle temperature and blood flow that rapidly increases within 3–5 min and reaches plateaus (Racinais et al., 2017), whereas the longer intra-complex rest intervals may result in its reduction. Moreover, type II fibers may especially benefit from the elevated muscle temperature leading to higher power outputs (De Ruiter and De Haan, 2000; McGowan et al., 2015).

A careful examination of the current body of scientific literature regarding the PAPE effect has yielded partially inconsistent findings. Wilson et al. (2013) concluded that the greatest performance enhancement is achieved after multiple sets of resistance exercises with longer intra-complex rest intervals (7–10 min), whereas Seitz and Haff (2016) suggest that rest intervals ≥5 min seem to be optimal. However, regarding the CA intensity, findings of Wilson et al. (2013) reported that a moderate intensity (60–84% 1RM) was more effective in inducing subsequent performance improvements than higher intensities (≥85% 1RM), whereas Seitz and Haff (2016) indicated that superior PAPE effect is reached after exercise intensity exceeds 85% 1RM. In regard to the differences of the obtained performance enhancement between the upper and lower limbs, a considerably superior effect was registered for the lower limbs by Wilson et al. (2013) (ES = 0.42 vs. 0.17) and by Seitz and Haff (2016) (for upper-body ballistic activities ES = 0.23 vs. for the jump ES = 0.31 and the sprint ES = 0.5), whereas results of this meta-analysis indicated even greater PAPE effect for the upper limbs (ES = 0.33). Nevertheless, the authors did not analyze the mechanisms responsible for these differences. Recently, more evidence has been presented confirming differences between upper- and lower-body PAPE responses. A study by Kilduff et al. (2007) found performance improvements in both upper- and lower-body explosive exercises after the same volume and intensity of the CA [single repetition of the BP or back squat at 3RM]; however, greater effects were observed for the lower limb exercise (5.3 vs. 8%). In turn, Farup and Sørensen (2010) reported no difference in BPT performance after five sets of 1RM BPs as the CA, whereas a back squat performed with the same variables elicited significant improvements in countermovement jump performance in a study by Gilbert and Lees (2005). Furthermore, Evetovich et al. (2015) directly compared post-partial back squat and post-BP shot put potentiation in male and female collegiate Division II athletes. The authors found that a shot put performance after a single set of the BP exercise at 3RM was significantly greater than that after partial back squats. Additionally, surprising findings have been provided by Cuenca-Fernández et al. (2017) who indicated that the PAPE effect could be elicited when the CA and subsequent movement involve different groups of muscles. A study by Cuenca-Fernández et al. (2017) revealed that a CA consisting of four BP repetitions at 90% 1RM improved squat jump performance among male and female swimmers; however, a control condition (standing quietly for 4 min) also led to significant enhancement. This may suggest that the improvement noted is more related to the quality of the warm-up, rather than the PAPE effect itself. Furthermore, the participants showed a low level of muscle strength (1.15 kg/bw for back squat and 0.91 kg/bw), which is known to have a significant impact on the magnitude of the PAPE effect. Therefore, based on these results, it can be speculated that optimizing the PAPE effect in the upper body requires a rather different approach than that in the lower body and, as this meta-analysis has shown, a slightly lower intensity than those proposed previously (Seitz and Haff, 2016).

The main limitation of the current meta-analysis is the small number of studies; however, the ones that were selected were homogenous. Moreover, nearly three-fourths of the studies were considered to be at moderate or serious risk of bias. Therefore, future studies should employ control condition trials, recruit more participants, provide a sample size power analysis, and measure the potentiation across several intra-complex rest intervals to increase the generalizability of results while reducing the risk of bias. Furthermore, studies in resistance-trained men were only included, and no subgroup according to the muscle strength level has been analyzed. Since there is a paucity of studies that directly compare the effects of the same CA on upper- and lower-body responses, there is a necessity of studies examining this topic. Consequently, it would be interesting to assess different CA approaches to clearly define the optimal range of the training variables to induce subsequent performance enhancement in resistance-trained men and women. What is more, the scope of research on the influence of the PAPE effect, undertaken recently, indicates that it may have a much wider application in training practice. Studies examining the effect of a CA on subsequent exercise volume (Alves et al., 2019; Krzysztofik et al., 2020a), the combined effects of supplements, and the implementation of blood flow restriction in order to increase the response (Wang et al., 2016; Guerra et al., 2018; Wilk et al., 2020) or the opposite approach of the potentiation complex (e.g., explosive task before resistance exercises with high intensity) (Wilcox et al., 2006; Krzysztofik and Wilk, 2020) should be continued.

## Conclusion

The present meta-analysis of the included studies indicates that performing a CA induces small PAPE effects in the BPT performance in resistance-trained men. Moreover, the obtained data suggest that the magnitude of enhancement depends on moderating variables of potentiation complexes, and that they should be considered collectively. These results may be of great practical significance for athletes and coaches of explosive sport disciplines, providing valuable information regarding the optimal range of each variable of potentiation complexes. Individuals seeking to acutely improve their BPT performance should consider preceding them with a single set of the BP exercise at moderate intensity (60–84% 1RM), performed 5–7 min before the explosive activity.

## Data Availability Statement

The raw data supporting the conclusions of this article will be made available by the authors, without undue reservation.

## Author Contributions

MK and MW: conceptualization. MK: data curation and formal analysis. MW and AG: supervision. MK: writing—original draft. MW, AG, and PS: writing—review and editing. All authors approved the final version of the manuscript to be submitted.

## Conflict of Interest

The authors declare that the research was conducted in the absence of any commercial or financial relationships that could be construed as a potential conflict of interest.
